# Proximal Femoral Nail versus Proximal Femoral Nail Antirotation: Functional and Radiological Outcome in Intertrochanteric Fractures of Femur

**DOI:** 10.7759/cureus.19093

**Published:** 2021-10-28

**Authors:** Siddhartha Singh

**Affiliations:** 1 Orthopaedics, All India Institute of Medical Sciences, Rae Bareli, Rae Bareli, IND

**Keywords:** osteoporotic, complications, neck shaft angle, tip apex distance, cleveland index, pps, hhs, intertrochanteric fractures, pfna, pfn

## Abstract

Introduction

Newer implant designs such as Proximal Femoral Nail (PFN) and Proximal Femoral Nail Antirotation (PFN-A) have shown promising results in the treatment of intertrochanteric fractures. Despite the availability of various implants for the treatment of these fractures, there is no common consensus as to which implant would be ideal. Therefore, there is a need for further clinical trials to establish the biomechanical and functional outcome superiority of implants such as PFN and PFN-A, especially among osteoporotic patients.

Aim

To compare the functional and radiological outcomes in intertrochanteric patients treated with PFN and PFN-A.

Methodology

A total of 152 intertrochanteric fracture patients were included in this retrospective study, 94 in the PFN group and 58 in the PFN-A group. The postoperative radiological outcome was assessed and compared using parameters such as tip-apex distance (TAD), Cleveland index, neck-shaft angle, and type of reduction. Operative time for the procedure, pre and postoperative hemoglobin levels were recorded and compared. Fracture union rates were compared at six weeks and six months. Functional outcome was compared between the two groups at follow-up period of 6 months using Harris Hip Score (HHS) and pre and postoperative Parker Palmer Mobility Score (PPS). Complication rates were compared between the two groups. Also, osteoporotic patients were evaluated using the same parameters.

Results

The radiological parameters were similar in both groups. There was a shorter operative time and better hemoglobin levels after surgery in the PFN-A group. The complication rate in the PFN group was 7.4% when compared to the PFN-A group which was just 1.7%. The functional outcome based on HHS and PPS was alike in the two groups. In osteoporotic patients randomized based on Singh’s index, better union rates were observed in the PFN-A group and a higher complication rate was seen in the PFN group.

Conclusion

Cephalomedullary nailing using PFN-A is superior to PFN in terms of a lesser procedure time, minimal blood loss, and fewer complications even among osteoporotic patients. The radiological specifications such as neck-shaft angle post-surgery, reduction type, TAD, and Cleveland index is of paramount importance which is established in this study.

## Introduction

Intertrochanteric fractures of the femur commonly occur in elderly osteoporotic individuals. Recumbency post hip fractures have been related to increased mortality among elderly patients. Surgical treatment is essential in such types of fractures for obtaining a reduction that is acceptable as well as for the early rehabilitation of the patients [1]. The dynamic hip screw (DHS) implant earlier considered to be the hallmark treatment for stable intertrochanteric fractures proved as inadequate for the fixation of the unstable type of fractures [2]. For the treatment of the unstable variety, a dynamic femoral head/neck stabilization implant coupled with intramedullary nailing is now considered to be ideal [3].

Numerous nail designs which incorporate a single compression screw or a compression screw coupled with antirotation screw such as Proximal Femoral Nail (PFN) are gaining popularity for the treatment of intertrochanteric fractures. Although PFN proved to have an upper hand when compared with extramedullary devices for unstable intertrochanteric fractures, screw cut-out, z effect, reverse z effect, and varus collapse accounted for 31% of the postoperative complications [4]. The Proximal Femoral Nail Antirotation (PFN-A) design increased the bone-implant interface as well as resulted in compacting the cancellous bone, henceforth giving a provision of excellent stability in terms of fixation [5]. The helical blade’s insertion which does not involve reaming out the bone from the head/neck fragment provides an additional anchorage especially among the osteoporotic age group of patients. Some studies prove that helical blade, by compacting the cancellous bone in its surrounding, provides better resistance to rotation as well as varus collapse [6].

However, there is a further need for clinical trials to confirm whether PFN-A is superior in functional and radiological outcomes. Limited studies on surgical procedures with PFN-A have shown that this implant can also be related with cut-through into the hip joint, back-out, and cut-out in similarity with the previous implants. Therefore, this study was performed for comparing the functional and radiological outcomes as well as the complications encountered with the use of PFN and PFN-A in the fixation of intertrochanteric fractures. The study also made an assessment of the comparative performance of the two implants in osteoporotic intertrochanteric fractures.

## Materials and methods

This was a retrospective study held in the department of orthopaedics of JSSAHER (JSS Academy of Higher Education and Research), Mysore, India. A total of 152 intertrochanteric fracture patients from February 2017 till February 2020 were included retrospectively in the study. The inclusion criteria were all intertrochanteric fracture cases classified on the basis of AO classification as well as skeletally mature patients of either gender who had undergone treatment with either PFN or PFN-A. The exclusion criteria were non-ambulatory patients before injury and patients with osteoarthritis of the hip before the injury. The AO (alphanumeric) classification was used for classifying the fractures based on preoperative anteroposterior (AP) and lateral radiographs of the affected side. Singh’s index was used for osteoporosis grading [7]. Preoperative and postoperative hemoglobin was recorded and the operative time was measured according to the anesthesia record sheet.

Assessment of the reduction quality was done by a comparison of the neck-shaft angle of the operated side and of the normal side on AP view of immediate postoperative radiographs. A variation of less than 5 degrees from the normal side was considered as a ‘good’ reduction. Between 5 and 10 degrees of variation was considered ‘acceptable’ and more than 10 degrees variation was considered ‘poor’ [8]. The quality of fixation was assessed using the tip-apex distance (TAD) described by Baumgaertner and the Cleveland index [9]. Measurement of the TAD was done using the Picture Archiving and Communication System (PACS) tool on the postoperative X-rays. Assessment of the compression screw position in PFN and helical blade position in PFN-A was done by using the Cleveland index. The placement of the compression screw or helical blade was considered to be acceptable when it was either centre-centre or centre-inferior.

Functional outcome was assessed by the pre and post-operative Parker Palmer Mobility Score (PPS) [10]. Comparison of the pre and postoperative scores was done at the final follow-up of 6 months. The Harris Hip Score (HHS) which was calculated at the final follow-up period of 6 months for assessing postoperative hip function was noted [11]. Any complications encountered during the follow-up period were noted for both groups of patients. Complications among patients with grade 3 and below Singh's index in both the groups also underwent comparison.

Statistics

The number of patients according to AO classification, age, and sex were evaluated by using proportions. The number of patients in each group (PFN and PFN-A) and operative time were evaluated using mean and standard deviation. Type of implant used according to AO classification and the type of reduction in both groups were evaluated using the Chi-square test. Neck shaft angle, TAD, Cleveland index, HHS, PPS, and complications in both the groups were evaluated using the independent t-test. A p-value of <0.05 was deemed to be of significance. A comparison of reduction in haemoglobin values between the two groups was done using the Mann-Whitney U test. SPSS Statistics 21.0 (IBM Corp, Armonk, USA) was used for all measurements.

## Results

A total of 152 patients with stable and unstable types of intertrochanteric fracture were included in the study. Ninety-four patients underwent treatment with PFN, whereas 58 patients with PFN-A. The majority of the patients (52%) were in the age group of 61-80 years, with 19% patients above 80 years and 29% below 61 years (Table 1).

**Table 1:** Distribution of patients based on age, sex, and classification type

		Count	%
Age group	<40	12	7.9%
	41-60	32	21.1%
	61-80	79	52.0%
	>80	29	19.1%
Sex	Female	68	44.7%
	Male	84	55.3%
AO	31-A1.1	1	.7%
	31-A1.2	3	2.0%
	31-A1.3	11	7.2%
	31-A2.1	14	9.2%
	31-A2.2	29	19.1%
	31-A2.3	36	23.7%
	31-A3.1	27	17.8%
	31-A3.2	24	15.8%
	31-A3.3	7	4.6%
Total		152

Male: Female ratio was 1:0.8. Based on the preoperative radiographs, 79% of patients belonged to the unstable group (AO 31-A2.2 to A3.3) whereas 21% of patients belonged to the stable group (AO 31-A1.1 to A2.1). In the PFN group, 82.9% of patients were in the unstable group whereas, in the PFN-A group, 77.5% of patients were in the unstable group (Table 2).

**Table 2:** Type of implant used in different classification groups PFN: Proximal Femoral Nail; PFN-A: Proximal Femoral Nail Antirotation

		Type of implant used			
		PFN		PFN-A	
		Count	%	Count	%
AO	31-A1.1	0	.0%	1	1.7%
	31-A1.2	3	3.2%	0	.0%
	31-A1.3	5	5.3%	6	10.3%
	31-A2.1	8	8.5%	6	10.3%
	31-A2.2	16	17.0%	13	22.4%
	31-A2.3	23	24.5%	13	22.4%
	31-A3.1	18	19.1%	9	15.5%
	31-A3.2	14	14.9%	10	17.2%
	31-A3.3	7	7.4%	0	.0%
Total		94		58

The neck-shaft angle (Table 3) was assessed in the immediate postoperative pelvis with bilateral hip radiographs (AP view). The values of both the groups were compared using the independent t-test and the difference was found to be insignificant (p=0.99).

**Table 3:** Assessment of neck-shaft angle among both the groups PFN: Proximal Femoral Nail; PFN-A: Proximal Femoral Nail Antirotation

	Group			
	PFN		PFN-A	
	Mean	Standard Deviation	Mean	Standard Deviation
Neck shaft angle (Degrees)	130.61	3.04	130.61	2.87

The type of reduction (Table 4) was compared among both groups. A positive reduction was seen in 59.6% of patients in the PFN group and 65.5% of patients in the PFN-A group. A neutral reduction was seen in 23.4% of patients in the PFN group and 24.1% of patients in the PFN-A group. A negative reduction was observed in 17% of patients in the PFN group and 10.3% of patients in the PFN-A group. On comparison using the Chi-square test, the result was found to be insignificant (p=0.52).

**Table 4:** Comparison of type of reduction among both the groups PFN: Proximal Femoral Nail; PFN-A: Proximal Femoral Nail Antirotation

		Group			
		PFN		PFN-A	
		Count	%	Count	%
Reduction	Negative	16	17.0%	6	10.3%
	Neutral	22	23.4%	14	24.1%
	Positive	56	59.6%	38	65.5%
Total		94		58

Harris Hip Score (Table 5) was compared among both the groups at a follow-up period of 9 months. For the PFN group, the mean score was 79.38 and for the PFN-A group the mean score was 79.62. The values were compared using the independent t-test and it was found to be insignificant (p=0.78).

**Table 5:** Comparison of HHS among the two groups HHS: Harris Hip Score; PFN: Proximal Femoral Nail; PFN-A: Proximal Femoral Nail Antirotation

	Group			
	PFN		PFN-A	
	Mean	Standard Deviation	Mean	Standard Deviation
Harris Hip Score	79.38	4.88	79.62	4.94

Postoperative Parker Palmer Mobility Score was compared between the two groups (Table 6). Its mean value in the PFN group was 7.41 whereas in the PFN-A group it was 7.09. The comparison was done using the independent t-test and was found to be insignificant.

**Table 6:** Comparison of postoperative PPS PPS: Parker Palmer Mobility Score; PFN: Proximal Femoral Nail; PFN-A: Proximal Femoral Nail Antirotation

	Group			
	PFN		PFN-A	
	Mean	Standard Deviation	Mean	Standard Deviation
Post op Parker Palmer mobility score (PPS)	7.41	.88	7.09	1.11

Operative time (Table 7) was compared among the two groups. The mean operative time for the PFN group was 47.98 and for the PFN-A group was 36.12. The difference was found to be significant (p<0.0001).

**Table 7:** Comparison of operative time PFN: Proximal Femoral Nail; PFN-A: Proximal Femoral Nail Antirotation

	Group			
	PFN		PFN-A	
	Mean	Standard Deviation	Mean	Standard Deviation
Operative time min	47.98	10.20	36.12	9.82

The two groups were compared based on the Cleveland index (Table 8). Centre-Centre (C-C) placement was seen among 48.9% in the PFN group and 39.7% in the PFN-A group. Centre-Inferior (C-I) placement was seen among 46.8% in the PFN group and 39.7% in the PFN-A group. The difference between the two groups was found to be insignificant.

**Table 8:** Comparison of the Cleveland index C-C: Centre-Centre; C-I: Center-Inferior; C-S: Center-Superior

		Group			
		PFN		PFN-A	
		Count	%	Count	%
Cleveland	C-C	46	48.9%	23	39.7%
	C-I	44	46.8%	31	53.4%
	C-S	4	4.3%	4	6.9%
Total		94		58

**Figure 1:**
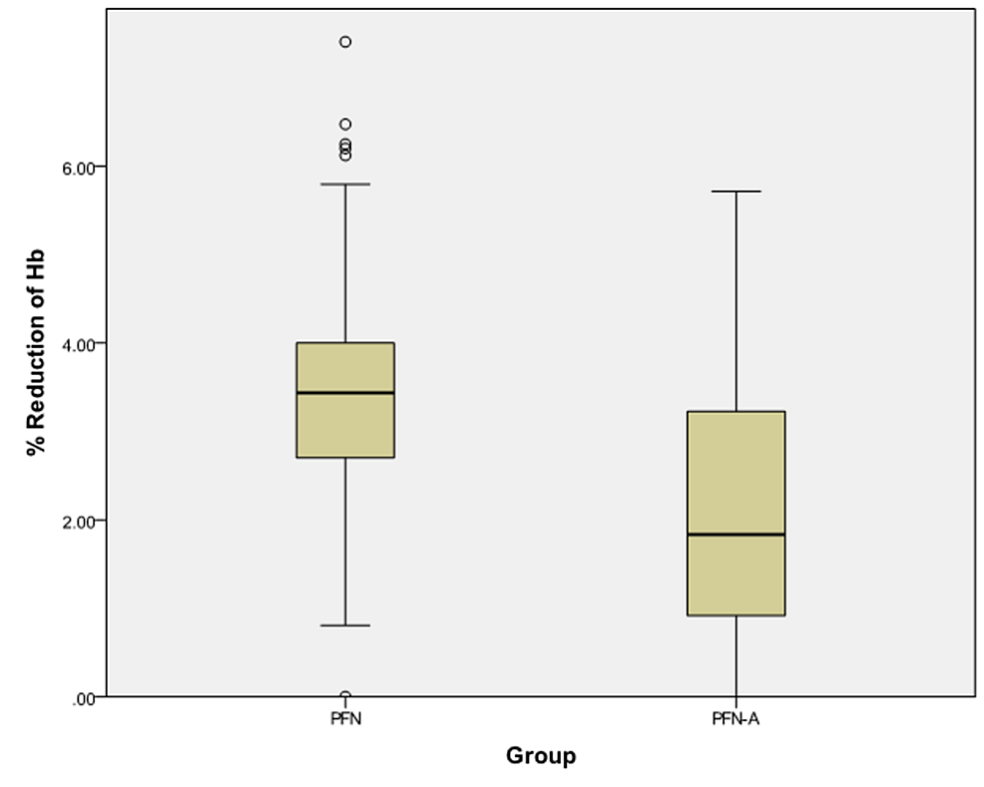
Percentage reduction of hemoglobin between the two groups Hb: hemoglobin; PFN: Proximal Femoral Nail; PFN-A: Proximal Femoral Nail Antirotation

The percentage of reduction of hemoglobin (Table 9, Figure 1) postoperatively was compared between the two groups using the Mann-Whitney U test, and the result was found to be significant.

**Table 9:** Comparison of reduction of hemoglobin values Hb: hemoglobin; PFN: Proximal Femoral Nail; PFN-A: Proximal Femoral Nail Antirotation

	Group						
	PFN			PFN-A			
	Median	Q1	Q3	Median	Q1	Q3	p
Reduction of Hb	.40	.30	.50	.20	.10	.40	<0.0001
% Reduction of Hb	3.43	2.70	4.00	1.84	.92	3.23	<0.0001

Complications (Table 10) were compared among both groups. The PFN group had total of seven cases: five cases of z effect, one case each of reverse z effect and screw back out. The PFN-A group had two complications: one case of implant breakage and one case of wound infection. PFN group had a complication rate of 7.4% whereas the PFN-A group had 3.4%. On comparing the results of the two groups, the p-value was found to be significant.

**Table 10:** Comparison of complications among the two groups PFN: Proximal Femoral Nail; PFN-A: Proximal Femoral Nail Antirotation

		Group			
		PFN		PFN-A	
		Count	%	Count	%
Complications	None	87	88.9%	56	100.0%
	implant breakage	0	.0%	1	.0%
	Reverse z effect	1	2.2%	0	.0%
	screw backout	1	2.2%	0	.0%
	wound infection	0	.0%	1	.0%
	z effect	5	6.7%	0	.0%
Total		94		58

The total number of osteoporotic patients (Singh’s index 1-3) in the PFN group was 45 and in the PFN-A group, it was 32 (Table 11). Both the groups among osteoporotic patients were compared on the basis of neck-shaft angle, TAD, type of reduction, HHS, PPS, and the number of complications.

**Table 11:** Comparison of Singh's index among osteoporotic patients PFN: Proximal Femoral Nail; PFN-A: Proximal Femoral Nail Antirotation

		Group			
		PFN		PFN-A	
		Count	%	Count	%
Singh’s index	Singh’s index (1-3)	45	47.9%	32	55.2%
	Singh’s index (4-6)	49	52.1%	26	44.8%
Total		94		58

The neck-shaft angle, TAD, HHS, PPS (Table 12), and the type of reduction (Table 13) among the two groups in osteoporotic patients were compared and the difference was found to be not significant.

**Table 12:** Comparison of neck shaft angle, HHS, PPS in osteoporotic patients among the two groups HHS: Harris Hip Score; PPS: Parker Palmer Mobility Score; PFN: Proximal Femoral Nail; PFN-A: Proximal Femoral Nail Antirotation

	Group				
	PFN		PFN-A		
	Mean	SD	Mean	SD	p
Neck shaft angle (Degrees)	130.74	3.64	130.82	2.22	0.9
Harris Hip Score	79.11	5.08	79.25	4.77	0.9
Post op Parker Palmer mobility score (PPS)	7.18	.72	6.47	.92	0.001

**Table 13:** Comparison of the type of reduction in osteoporotic patients among the two groups PFN: Proximal Femoral Nail; PFN-A: Proximal Femoral Nail Antirotation

		Group			
		PFN		PFN-A	
		Count	%	Count	%
Reduction	Negative	11	24.4%	3	9.4%
	Neutral	10	22.2%	6	18.8%
Positive		24		23

The PFN group in the osteoporotic patients had five complications whereas in the PFN-A group no complications were seen (Table 14). The difference between the two groups was found to be significant.

**Table 14:** Comparison of complications in osteoporotic patients PFN: Proximal Femoral Nail; PFN-A: Proximal Femoral Nail Antirotation

		Group			
		PFN		PFN-A	
		Count	%	Count	%
Complications	None	40	88.9%	32	100.0%
	implant breakage	0	.0%	0	.0%
	Reverse z effect	1	2.2%	0	.0%
	screw backout	1	2.2%	0	.0%
	wound infection	0	.0%	0	.0%
	z effect	3	6.7%	0	.0%
Total		45		32

Clinical cases

Case 1

**Figure 2:**
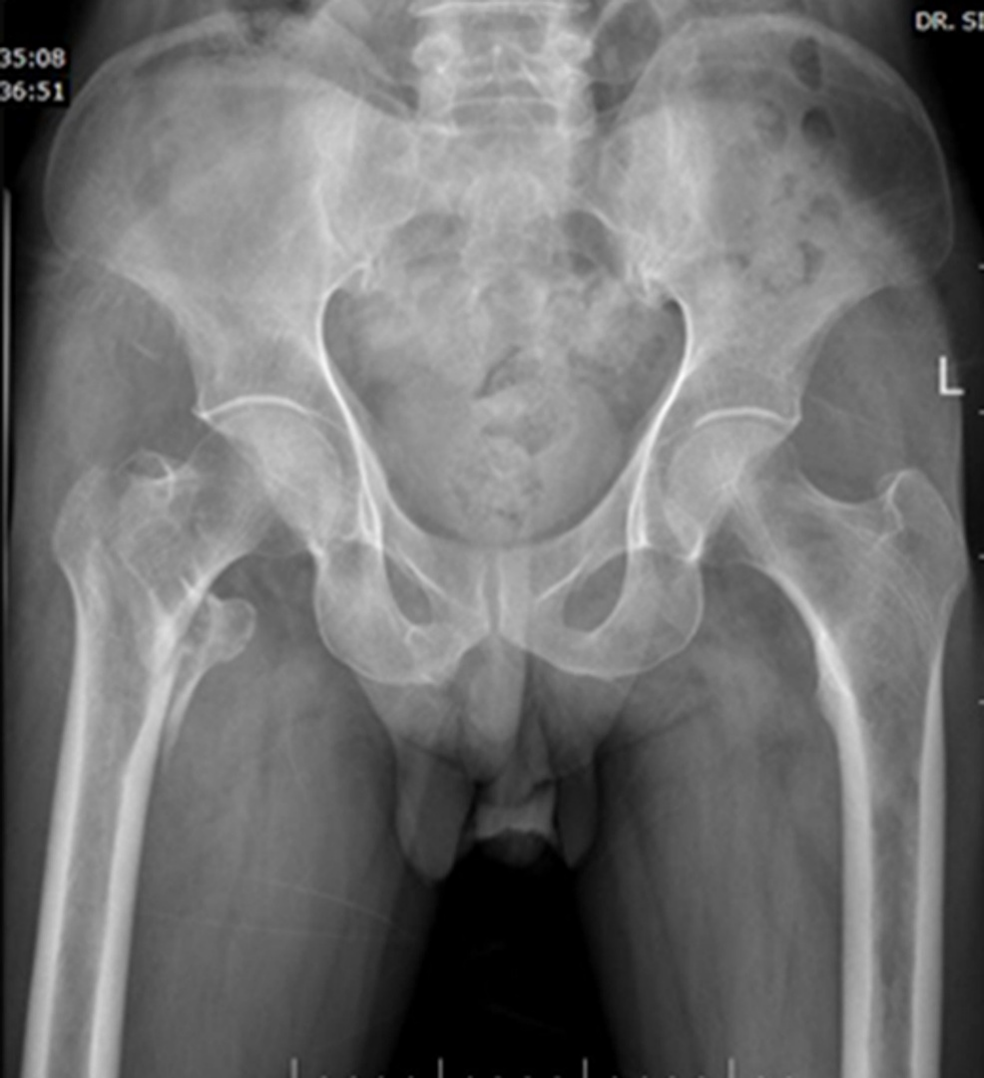
Preoperative pelvis with bilateral hip - AP view AP: anteroposterior

**Figure 3:**
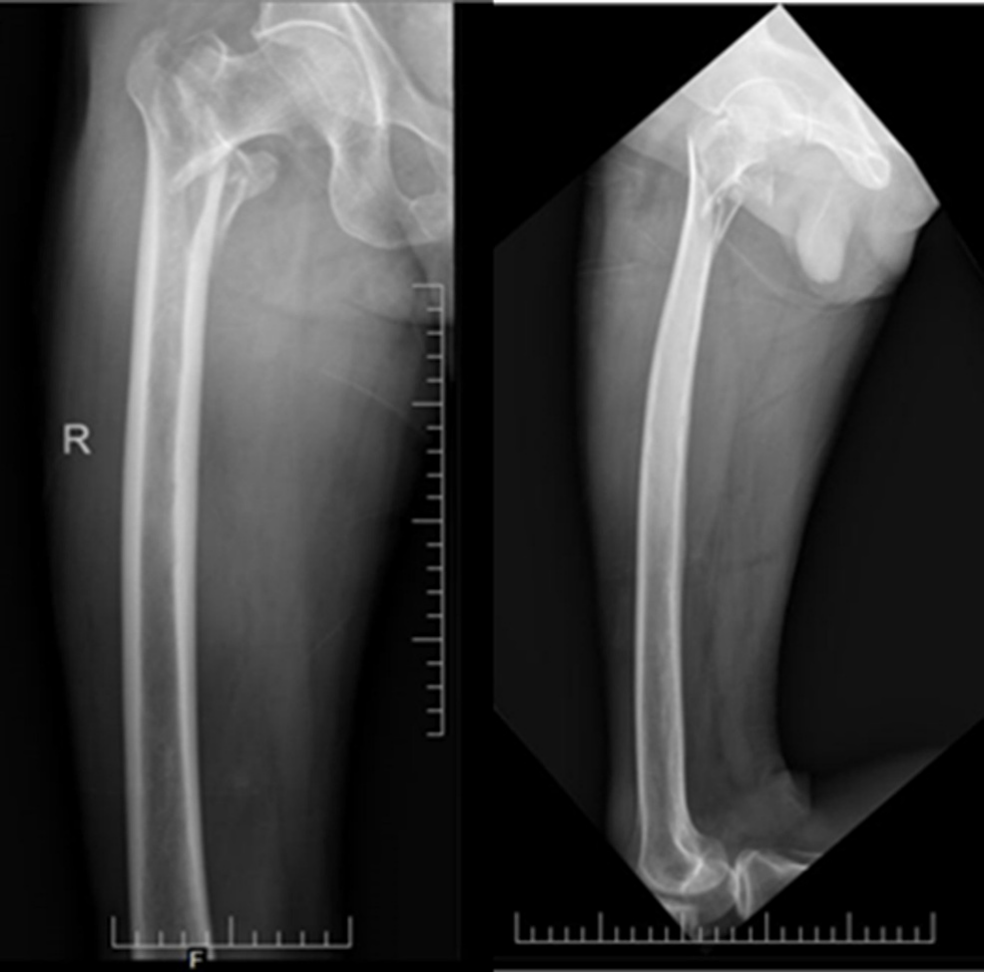
Right hip radiograph - AP (L) and lateral (R) views

A 67-year-old male patient sustained an injury over the right hip following a fall from stairs. Pelvis with bilateral hip-AP X-ray (Figure 2) and right hip AP and lateral views (Figure 3) confirmed intertrochanteric fracture (AO31-A2.2).

**Figure 4:**
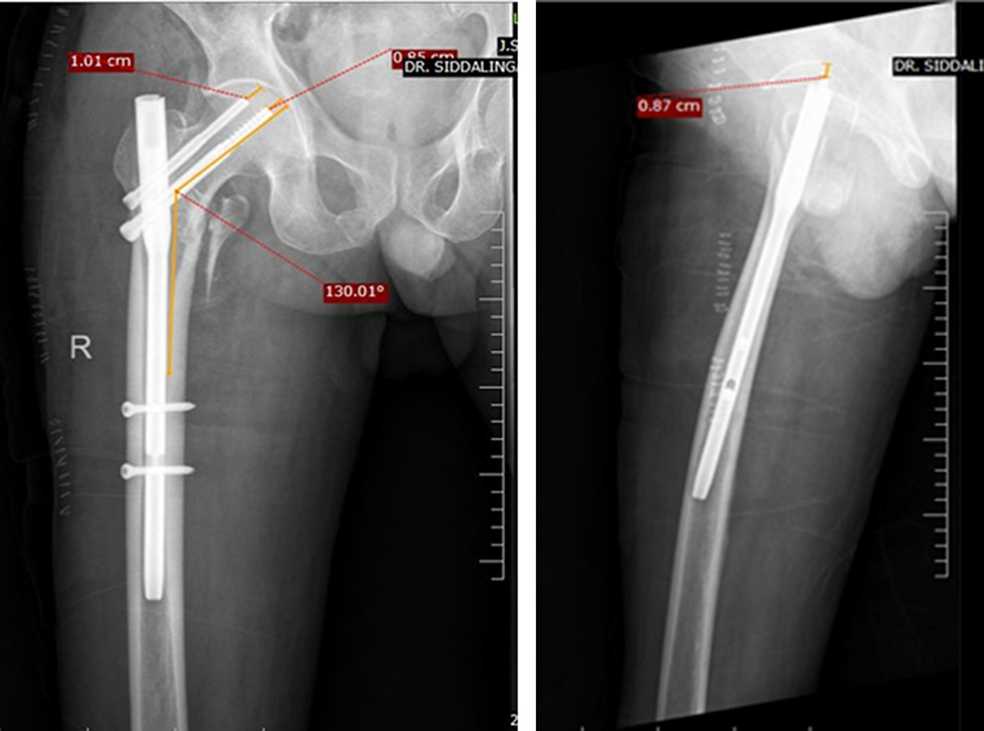
Measurement of radiological parameters- Right hip AP (L) and lateral (R)

The patient underwent closed reduction and internal fixation (CRIF) with PFN. Immediate post-operative X-rays showed adequate reduction by the measurement of neck-shaft angle, TAD (Figure 4), and the Cleveland index. Also, the type of reduction was noted to be positive.

**Figure 5:**
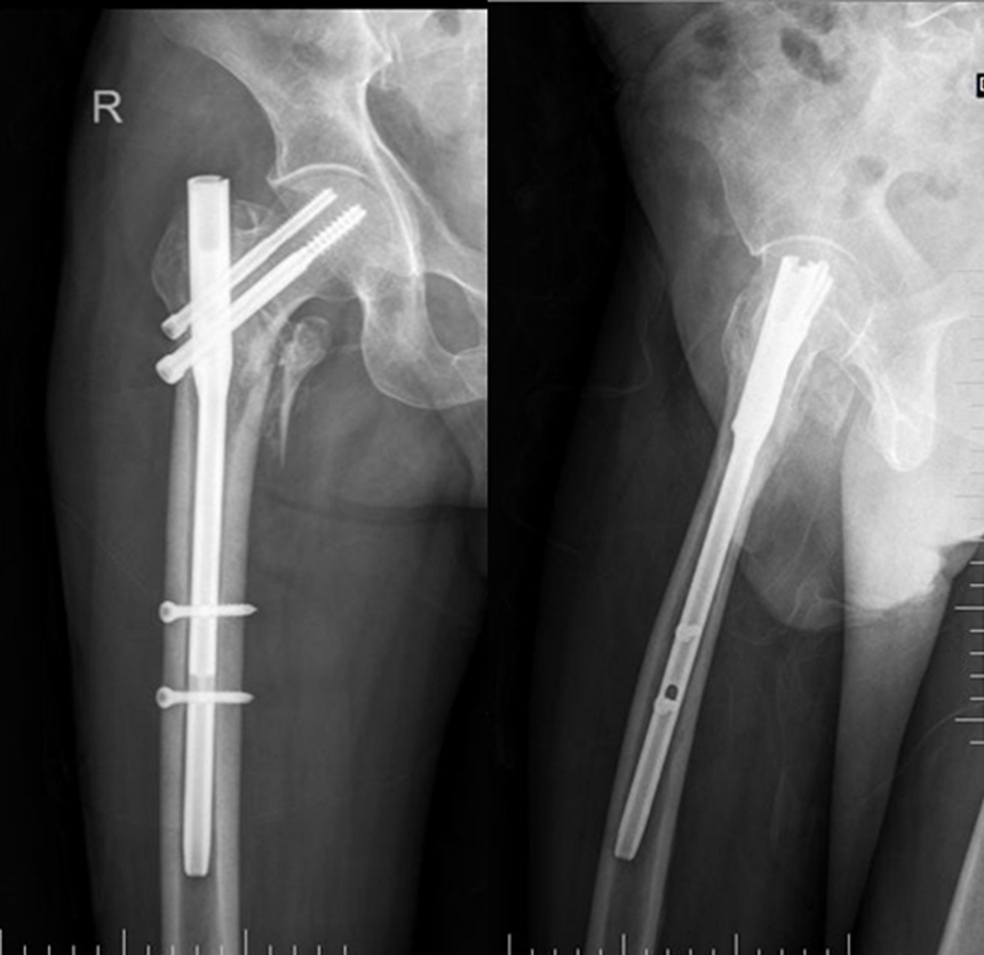
Six weeks follow-up - right hip AP (L) and lateral (R) radiographs AP: anteroposterior

The six-week follow-up X-rays showed adequate radiological outcome in terms of maintenance of the neck-shaft angle, TAD, and radiological signs of healed fracture (Figure 5). Final follow-up was done for the patient at 6 months where the hip range of motion (ROM), HHS, and post-op PPS were assessed, which were found to be in the satisfactory range.

Case 2

**Figure 6:**
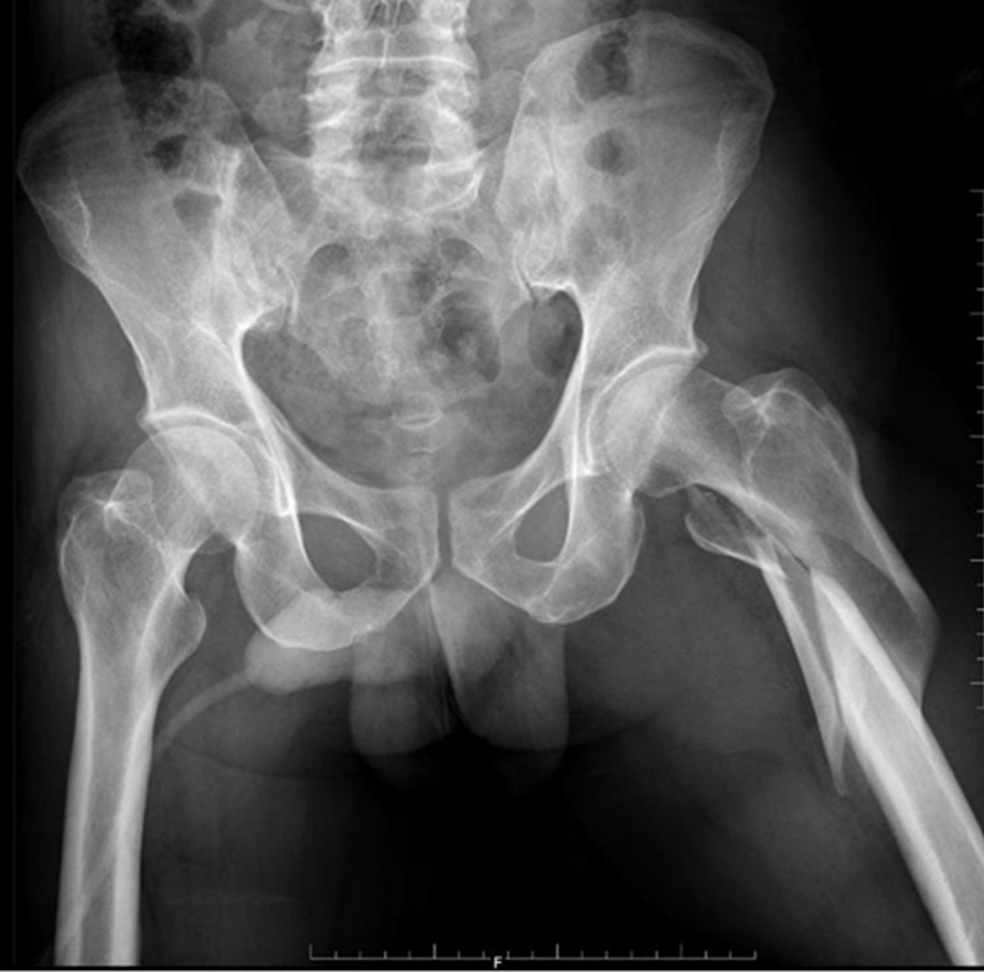
Pre-operative pelvis with bilateral hip - AP radiograph AP: anteroposterior

A 47-year-old female patient sustained an injury to the left hip following a road traffic accident (RTA). Pre-operative radiographs of the pelvis with bilateral hip - AP view (Figure 6) showed unstable intertrochanteric fracture (AO 31-3.3). The patient underwent CRIF with long PFN-A.

**Figure 7:**
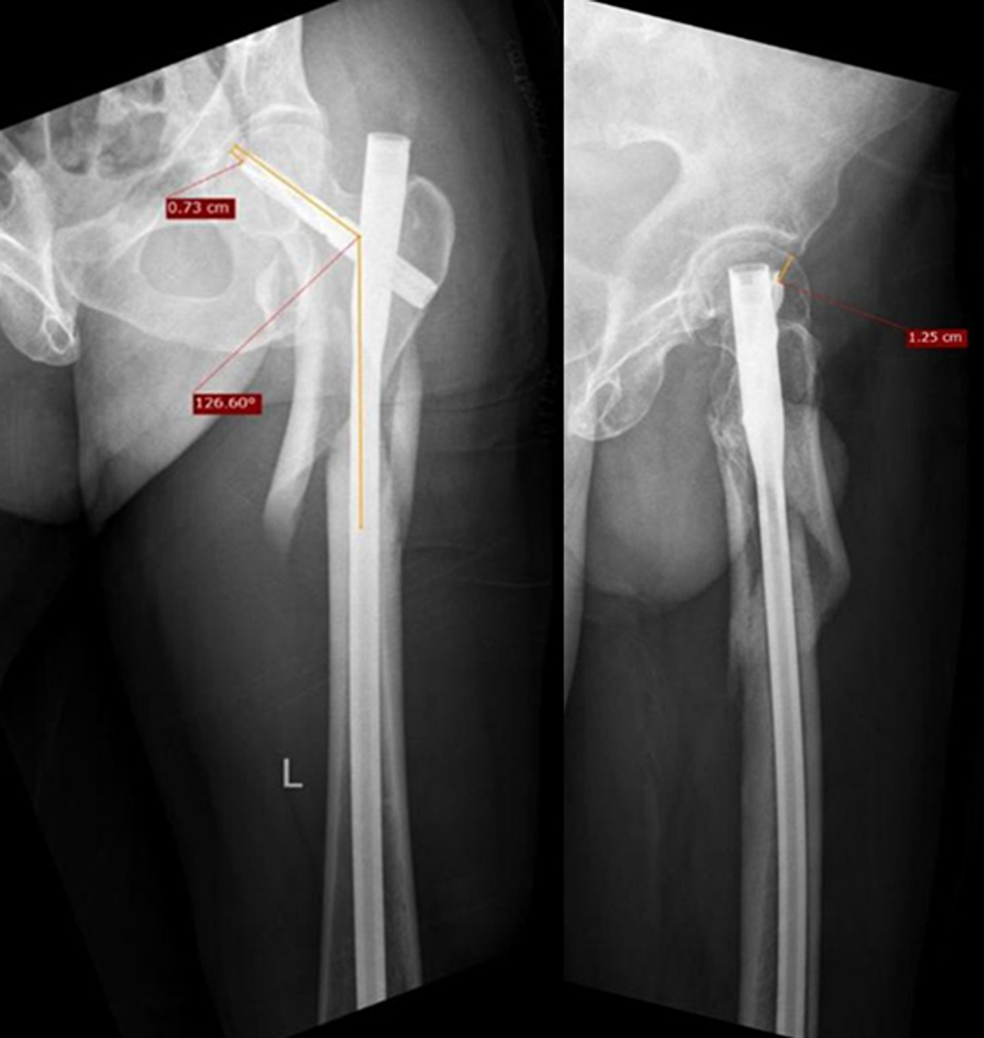
Measurement of postoperative radiological parameters- Left hip AP (L) and lateral (R)

**Figure 8:**
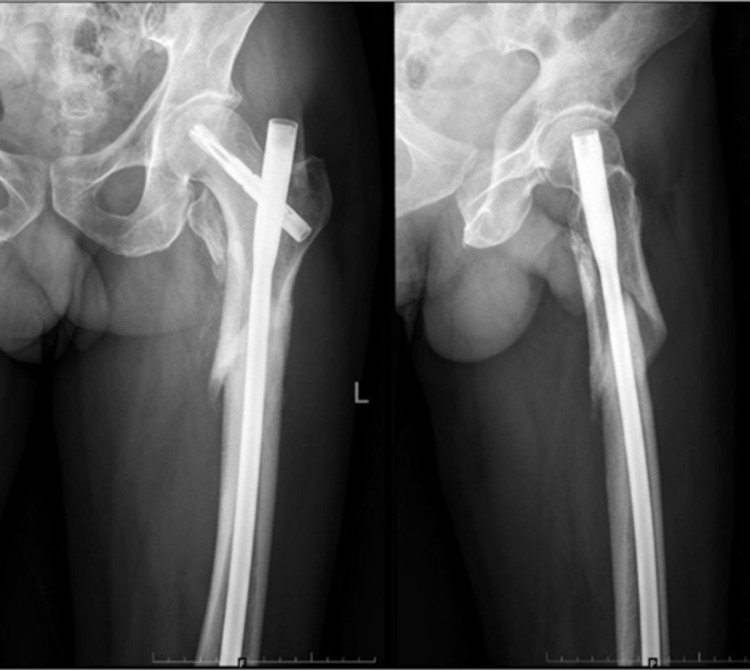
Six weeks follow-up left hip - AP (L) and lateral (R) radiographs AP: anteroposterior

**Figure 9:**
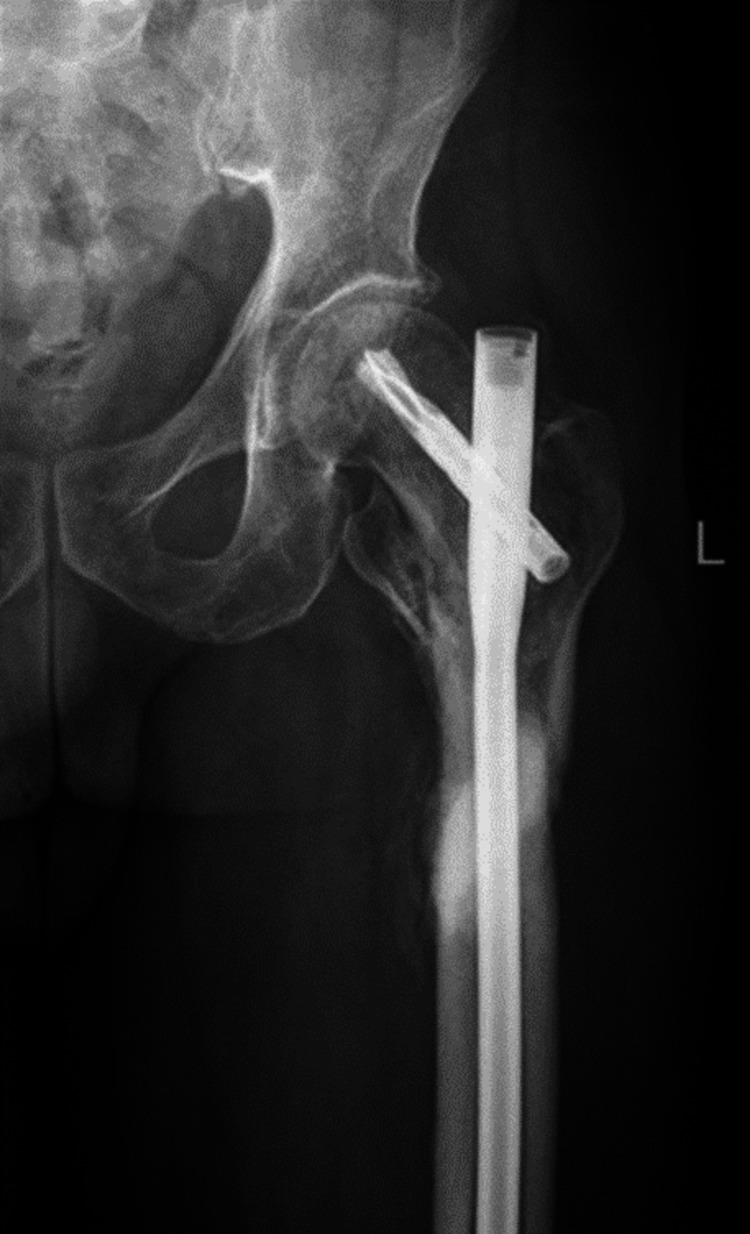
Six months follow-up radiograph

Immediate postoperative radiographs were assessed for the type of reduction, Cleveland index, TAD, and neck-shaft angle (Figure 7). Radiographs taken at six-week follow-up (Figure 8) showed signs of union at the fracture site with adequate radiological parameters which were further maintained in the radiographs taken at the final follow-up of 6 months (Figure 9). Functional outcome was assessed at the final follow-up of 6 months in terms of hip ROM, HHS, and post-op PPS which were found to be in the satisfactory range.

Complications

Case 1

**Figure 10:**
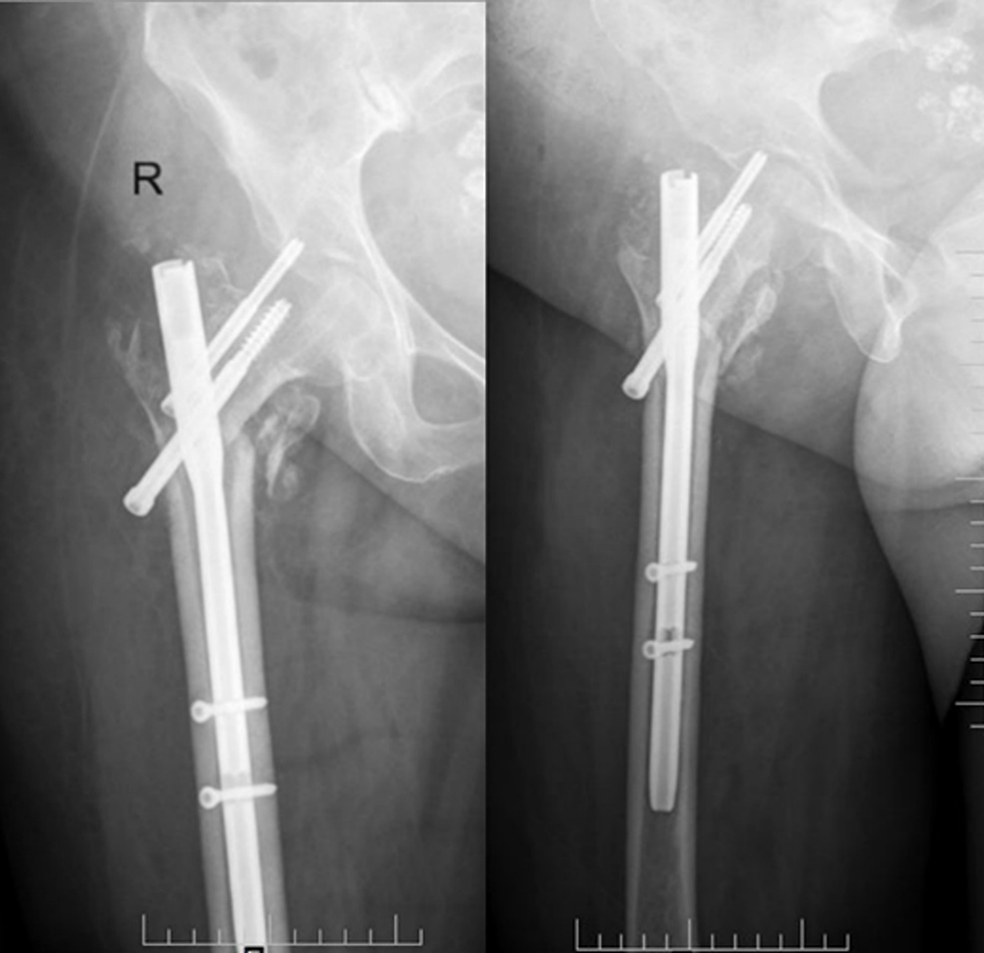
Z effect at two-month follow-up- Right hip AP (L) and lateral (R)

A 70-year-old male patient who had undergone CRIF with PFN came to the outpatient department two months after surgery with the chief complaints of pain in the right hip and difficulty in walking. Pelvis with bilateral hip - AP view radiograph showed complication in the form of z effect (Figure 10). The patient was then managed with hemiarthroplasty with bipolar. The patient had sustained AO 31-2.3 type of right-sided intertrochanteric fracture for which CRIF with PFN was done. Important radiological parameters which must be noted in the post-op radiograph are the type of reduction (negative), neck-shaft angle (120 degrees), and TAD (34.3 mm) which may have played a role in implant failure.

Case 2

**Figure 11:**
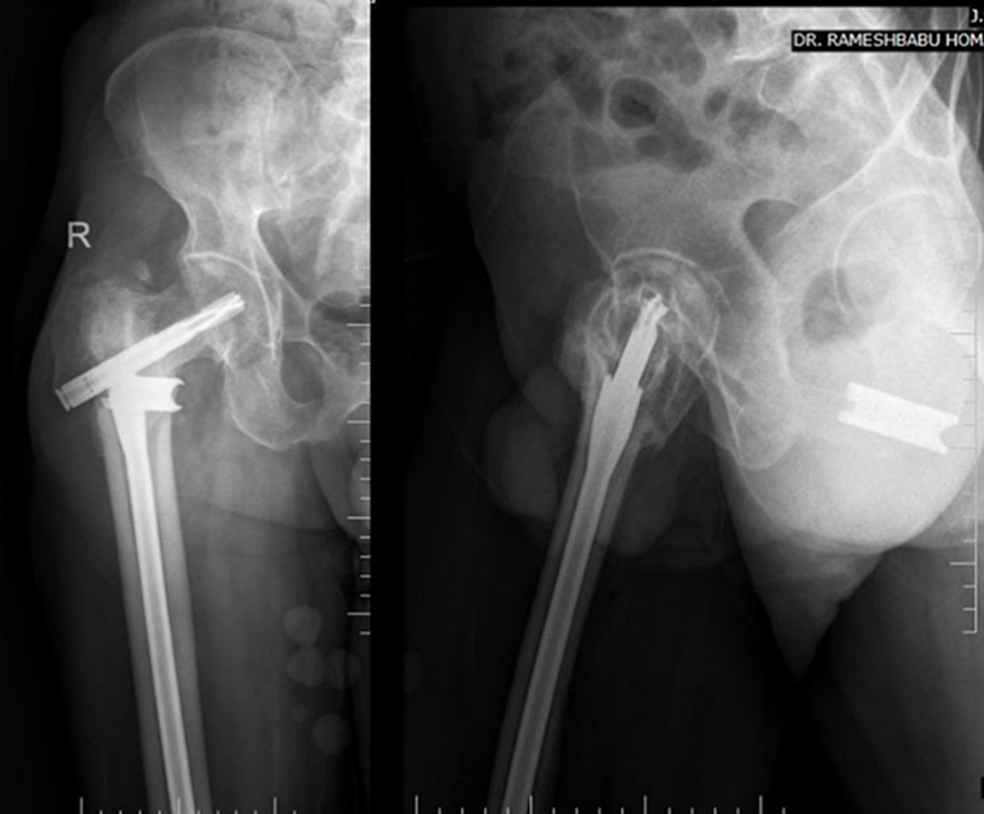
Five months follow-up - implant breakage- Right hip AP (L) and lateral (R)

A 67-year-old male patient came 5 months after surgery to the OPD with the chief complaints of pain in the right hip following an RTA. Radiographs following the injury showed implant breakage (Figure 11). Five months back, the patient had sustained AO 31-2.3 type of intertrochanteric fracture for which he had undergone CRIF with long PFN-A. Postoperative radiographs showed adequate radiological parameters in the form of TAD (17.4mm). However, the implant had a slight varus angulation.

## Discussion

In this study, the functional and radiological outcomes WERE compared between the PFN and PFN-A groups of patients based on various parameters such as operative time, preoperative and postoperative hemoglobin levels, Singh’s index, neck-shaft angle, reduction type, Tip Apex Distance (TAD), Cleveland index, HHS, and pre and postoperative PPS.

A total of 152 intertrochanteric fracture patients who were treated either with PFN or PFNA were included in this study retrospectively. Ninety-four patients belonged to the PFN group, whereas 58 patients belonged to the PFN-A group. The sixty-one to 80 years age group category included the highest number of patients resembling the etiology of intertrochanteric fractures. The mean age for the PFN patients was 64 years and PFNA patients were 68 years. There were 84 male patients and 68 female patients, which was in the ratio of 1.2:1.

According to the alphanumerical system (AO classification), the majority (23.7%) of the total patients belonged to 31-A2.3. This study includes stable as well as unstable intertrochanteric fractures, which has not been done in previous studies. Most of the previous studies done on intertrochanteric fractures evaluated the functional outcome of the implant used in unstable fractures. As a result, this study would shed light on the functional outcome of PFN and PFNA in all types of intertrochanteric fractures, whether stable or unstable, and hence, provide a better insight into which type of implant would be best suited for a patient with intertrochanteric fracture.

Hemoglobin values were tested preoperatively to determine intraoperative blood loss and the general nutritional status of the patient. Postoperatively, the blood loss was quantified by measuring the postoperative hemoglobin levels.

Based on the above values, it was noted that the PFN group of patients had slightly lower postoperative hemoglobin levels in comparison with the PFNA group. This difference was found to be significant and it provides us with an indirect insight into the amount of blood loss that would have occurred during the procedure of both the implant groups. Thereby, indicating that the amount of blood loss was less in the patients who underwent intertrochanteric fixation with PFNA. This can be attributed to the fact that lesser operative time was required for the PFNA procedure. However, a better estimation of blood loss could have been the measurement of pre and postoperative hematocrit levels, which can be done in future studies.

The majority of the operative procedures were finished between 30 minutes to 1 hour. A statistically significant difference was noted in the operative time in the PFNA group when compared to PFN group. Sharma et al's study also concluded that surgery time required for PFN-A was less in comparison to PFN [12]. Kashid et al's study also revealed that the operative time was lesser in the PFNA group, which resembles the data of this study [13].

In this study, the maximum number of patients (95.7%) had a satisfactory Cleveland index [9]. In the PFN group, a greater number of patients had the compression screw at centre-inferior (46.8%) and centre-centre (48.9%) position, whereas in the PFNA group 53.4% had centre-inferior and 39.7% had centre-centre position of the helical blade. In Sharma et al's study, 83% of PFN patients and 68% of PFNA patients had optimal positioning of the implant [12]. In this study, the positioning of the implant was better with 95.7% in the PFN group and 93.1% in the PFN-A group. Also, it was revealed that the implants having a centre-superior position suffered from more complications in comparison with the implants which had an optimum positioning of the compression screw/helical blade. This observation is of importance since it has not been observed in previous similar studies.

In the study, it was found that a large number of patients had HHS between 80 to 90 which signifies a good grade. Also, the mean HHS was similar in both the groups; 79.4 for PFN and 79.6 for PFN-A. This sheds light on the fact that both the implants have similar efficacy in terms of functional outcomes. The study done by Kashid et al reveals similar results in terms of HHS among both groups of patients [13]. Also, the difference in the post-surgery PPS between both the groups was not significant, pointing out to a similar functional result. 

As a part of the secondary objective of the study, the efficacy of both the implants among osteoporotic patients was compared. Superior union rates were observed during the follow-up period of six weeks and six months among patients with osteoporosis (Singh’s index grade 1-3) who had PFNA implants. Thereby suggesting that PFNA provides a better radiological outcome in comparison with PFN among patients with osteoporosis.

In the study, a greater number of complications were observed among the PFN group: 92.6% of the PFN patients had no postoperative complications. Out of the 7.4% who had complications, 1.1% had screw backout, 1.1% had reverse z effect and 5.3% had z effect. 96.6% of PFNA patients had no postoperative complications. 1.7% had implant breakage and 1.7% had post-operative wound infection. The majority of the patients having complications had neck-shaft angles less than 125 degrees. Also, a negative reduction was observed in the majority of the patients having complications. The TAD was found to be more than 25 mm in most of the patients who had complications. The position of the compression screw/helical blade was found to be centre-superior in the patients having complications. Insufficient radiological parameters might have led to the impending failure of the implant in patients who had complications. Thereby, implying the importance of the above radiological parameters for the success rate of PFN and PFNA. Sharma et al's study [12] also concluded with a greater complication rate among the PFN patients. However, the reason behind the high complication rate was not investigated in that study. A study done by Gardenbroek et al also observed a higher number of complications in the PFN group [14]. In contrast to this study, Kashid et al found that the complication rate among the two groups was not statistically different [13]. However, the number of patients included in that study was too less to obtain a definitive conclusion.

In this study, it was found that the majority of the patients had positive reduction (61%) with 23% of the patients having neutral reduction. The remaining patients had a negative reduction and it was observed that the patients in this group had a higher complication rate.

The neck-shaft angle was in the range of good reduction (61.8%) and acceptable reduction (23.7%) for the majority of the patients in the study. There were few patients with poor reduction (14.5%). A similar neck-shaft angle was observed among both groups. Complication rates were observed to be higher in patients with neck-shaft angles in the unsatisfactory range. Thereby, implying the need for an adequate neck-shaft angle post-surgery, which would lead to better results.

The mean TAD in the PFN group was more in comparison with the PFNA group. However, the difference in the values was insignificant. Sharma et al's study also concluded with similar TAD values among both the groups [12].

This particular study is unique as it reveals the need for satisfactory radiological parameters to reduce the number of complications. It sheds light on the paramount importance of the neck-shaft angle, type of reduction, and position of the compression screw/helical blade post-surgery. Also, it showcases a higher union rate among patients with osteoporosis operated upon with PFNA. This can help surgeons in decision-making for osteoporotic patients. In this study, all types of intertrochanteric fracture patients were included, which is not seen in the previous studies. The number of included patients is large enough to provide a better insight into the merits and demerits of both types of implants.

## Conclusions

In conclusion, PFNA shows superiority over PFN as it has a lesser procedure time, blood loss, and complication rate and has a better outcome among intertrochanteric fracture patients with osteoporosis.
